# MicroRNA regulation of molecular pathways as a generic mechanism and as a core disease phenotype

**DOI:** 10.18632/oncotarget.2734

**Published:** 2015-01-22

**Authors:** Rotem Ben-Hamo, Sol Efroni

**Affiliations:** ^1^ The Mina and Everard Goodman Faculty of Life Science, Bar Ilan University, Ramat-Gan, 52900, Israel

**Keywords:** microRNA, Signaling pathways, Cancer, Phenotype, Regulation

## Abstract

The role of microRNAs as key regulators of a wide variety of fundamental cellular processes, such as apoptosis, differentiation, proliferation and cell cycle is increasingly recognized in most aspects of biology and biomedicine. Accretion of results from multiple microRNA studies over multiple pathway networks, led us to hypothesize that microRNAs target molecular pathways. As we show here, this is a network-wide phenomenon. The work presented, uses statistical tools that show how single microRNAs target molecular pathways. We demonstrate that this targeting could not be the result of random associations and cannot be the result of the sheer numeracy of microRNA targets. Furthermore, the strongest evidence for the association microRNA-pathway, is in a demonstration of the way by which these associations are disease-relevant. In our analyses we study ten different types of cancer involving thousands of samples, and show that the identified microRNA–pathway associations demonstrate a clinical affiliation and an ability to stratify patients. The work presented here shows the first evidence for a mechanism of microRNAs-pathway generic regulation. This regulation is tightly associated with clinical phenotype. The presented approach may catalyze targeted treatment through exposure of hidden regulatory mechanisms and a systems-medicine view of clinical observation.

## INTRODUCTION

MicroRNAs (miRNAs) are small, endogenous non-coding RNA molecules that control gene-expression by inhibiting translation or inducing cleavage of target mRNAs. The role of miRNAs as key regulatory molecules that control a wide variety of fundamental cellular processes, such as proliferation, death, differentiation, motility, invasiveness, etc., has been demonstrated [1, 2]. MiRNAs are aberrantly expressed in cancer tissues and the connection between deregulated miRNAs and the inhibition of tumor suppressor genes in cancer is well established [3, 4]. Further, several studies have demonstrated a potential utility of miRNA-based therapy in cancer [5–8]. A striking example is the use of anti-miR-21 in breast cancer, which led to suppression of both cell growth *in vitro*, as well as tumor growth *in vivo* [9]. MiRNAs’ potential to act both as therapeutic agents and as disease biomarker places this family of non-coding RNAs at the forefront of biomedical interest. Cellular function and cellular pathways are thus affected by the regulatory function of miRNAs. The most studied of these processes include development, apoptosis, differentiation, and other oncogenic related processes [10]. A possible explanation for the dominating influence of miRNAs might therefore be the control, by a single miRNA, over an intricate pathway, through targeting multiple mRNAs of this specific cellular pathway. It is recognized, that one miRNA may be simultaneously targeting several mRNAs. These mRNAs could be members of a cascade functioning towards a functional endpoint in the cell, through mutual involvement in the same cellular signaling pathways or in the crosstalk between such pathways [11]. For example, miR-106a directly down regulates ULK1 mRNA levels in acute myeloid leukemia (AML) cells, and can also target other members of the ULK1 complex such as mAtg13 and FIP200 [12]. Other examples include Lu *et al.* [13] demonstration of microRNA-21 as down regulating the IL-12/IFN-γ pathway in lung cancer, and a work describing microRNA-7 as targeting 3-kinase/Akt pathway in hepatocellular carcinoma and Glioblastoma [14, 15]. Multiple targeting may be considered in view of how microRNA-200 functions as a multifunctional tumor suppressor in meningiomas through multiple, simultaneous, effects on the E-cadherin and Wnt/β-catenin signaling pathways [16]. The Pten/Akt pathway demonstrated inhibition in response to microRNA-1 [17] while the C/EBP-α–PU.1 pathway has been suggested to be regulated by microRNA-124 [18]. These important studies conceptually converge to raise the question of generality of this phenomenon: what is the breadth of this biological effect?

To answer this question, we analyzed a comprehensive collection of 357 pathways (137 NCI/Nature curated pathways and 220 pathways imported from the Biocarta database) from the National Cancer Institute's Pathway Interaction Database (PID) [19], which together comprise of 1460 genes. Using information on the complete collection of documented miRNAs - their predicted and validated target genes - we combined miRNA data and pathway data to identify, for each pathway, a single miRNA that potentially targets the pathway. The decision is based on statistical methods described below, and initially we demonstrate, per each miR-pathway, that this targeting is statistically significant. Following these results and utilizing them, we follow relations between identified miRNAs and their pathway targets and show that this targeting is instrumental in phenotypic stratification over a large collection of different cancer types.

As mentioned above, a critical role for miRNAs in cancer has been established. Further, multiple targeting of mRNAs, by a single miRNA, has also been universally demonstrated; the transition from multiple, disengaged, mRNA targets, to an hypothesis about miRNA and functional pathways has been raised, but has not yet been demonstrated. Using computational methods, we reveal significant associations between known curated pathways and specific miRNAs. We also show that this association is unique only to pathways. It is absent from otherwise dissociated random gene groups. Furthermore, and most important from a translational point of view, we show how these miRNA-Pathway associations are correlated with disease phenotypes, demonstrated in multiple datasets from ten different cancer types.

## RESULTS

### Pathways are targeted by individual miRNAs

**Figure 1:**
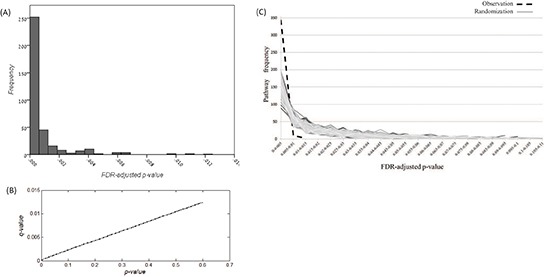
The observed enrichment of pathway targeting by miRNAs **(A)** Pathways FDR-adjusted *p*-value distribution across observed data. The figure presents a histogram of the number of targeted pathways, as a function of their FDR-adjusted *p*-values. All of the pathways show an (adjusted) *p*-value of lower than 0.014. **(B)** *p*-value vs. *q*-value (FDR-adjusted *p*-value) plot. The plot presented here shows the significance of the results. **(C)** By randomizing gene groups, we measure the uniqueness of naturally observed findings displayed in panel (A). The figure shows the same histogram as the one presented in panel (A), with results from randomized by selecting pathway-sized groups from the pool of genes that are members of any pathway. As the figure shows, over 1000 iterations present a clear separation from computationally observed findings with a significantly higher FDR-adjusted *p*-values (*p*-value = 3.35e-037).

We analyzed 357 curated pathways from the PID database [19] with overall 1460 genes. Initially, to partner these data with miRNA information, we used 8 prediction tools collectively combined into miRWalk [20]. miRWalk is a tool that performs a comparative analysis of predicted and validated targets through the combined use of 8 prediction tools: miRnada, miRDB, miRWalk, RNAhybrid, PICTAR5, PITA, RNA22, TargetScan. From these results, we chose the set of pairs of miRNA-Gene that were predicted by at least two different tools. We then tagged each gene with its affiliated pathway and further affiliated, with every pathway, a single miRNA. This single miRNA has been chosen as the miRNA with the highest number of targets in that specific pathway (results shown in Supplementary Table 1). A single *p*-value has been associated with every pathway and it's indicated miRNA. This *p*-value has been obtained using enrichment considerations, utilizing the hyper-geometric function (see Methods). All of the 357 pathways presented a significant FDR-adjusted *p*-value as can be seen in Figure 1(A) and Figure 1(B). FDR *p*-values were calculated using the procedure introduced by Storey, 2002 [21] for the correction of the *p*-values received by the hyper-geometric function.

Instead of setting the error rate at a particular level and estimating the rejection region, Storey et al. have proposed to fix the rejection region and estimate the error rate. This approach allows a more straightforward analysis of the problem.

Under multiple hypotheses testing, FDR procedures are designed to control the expected proportion of “false discoveries”.

To determine the strength and accuracy of the overall performance of the miRNA-pathway association using bootstrapping, we scrambled the data and randomly build 357 new pathways with a random size between 4–50 genes (as in the original data).

**Figure 2:**
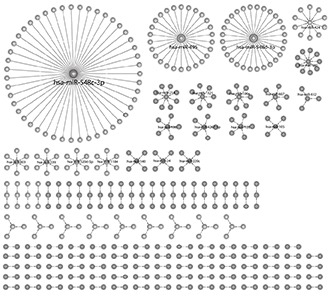
The network created from the analysis Every node represents either a pathway or a micro-RNA, edges represents the association between a micro-RNA and a pathway. The network composed out of 220 nodes and 186 edges and was visualized by Cytoscape.

Thus, we create an estimator for the hypothesis in order to test the hypothesis distance from a random, non-pathway related, distribution. Figure 1(C) shows both the observed FDR distribution and random FDR distribution across 1000 iterations. The results presented here suggest that the observed phenomenon of miRNA-pathway association is indeed specific (and highly significant with *p*-value of 3.35e-037) to defined cellular networks and is not observed in a non-biologic random network. In addition, this analysis revealed that there are 4 microRNA (hsa-miR548c-3p, hsa-miR-548d-3p, hsa-miR-495, hsa-miR-424) that are associated with 34% of the pathways (Supplementary Figure 1). Figure 2(A) presents the network created by these associations and Figure 2(B) shows the largest sub-network, composed of targets of hsa-miR-548c-3p.

### GATA3 pathway in targeted by hsa-miR-532 in ER- breast cancer patients

To investigate if these findings – the association between a specific miRNA and a specific pathway – have biological meaningful implications, we used methods that quantify network behavior from gene expression data. To utilize these network graph structures and the overlay of transcriptional data, we used the methods described in [22, 23]. These methods quantify expression behavior in specific sub-networks (i.e. specific pathways or any other defined sub-network) and produce metrics of network behavior and disruption. The analysis takes into consideration the specific type of interaction (such as inhibition or promotion) and calculates the likelihood that the interaction occurs in the pathway Further details are given in [22, 24]. To apply this network-based methodology, we used the tool PathOlogist [25] which is an automated tool that uses gene-expression data (RMA levels) to deduce pathway metrics. Each sample was thus presented by its pathway metrics.

To utilize this hypothetical miRNA-pathway regulation in understanding disease mechanisms, we used three different breast cancer datasets for the purpose of finding a consistent and robust pathway that stratifies patients into two groups, based on their Estrogen Receptor (ER) status. Estrogens are important regulators of growth and differentiation in the normal mammary gland and are also important in the development and progression of breast carcinoma. Estrogens regulate gene expression via ER, however the details of estrogen effect on downstream gene targets, the role of cofactors, and cross-talk between other signaling pathways are far from fully understood [26–29]. The expression of ER has important implications in therapy [30–32].

**Figure 3:**
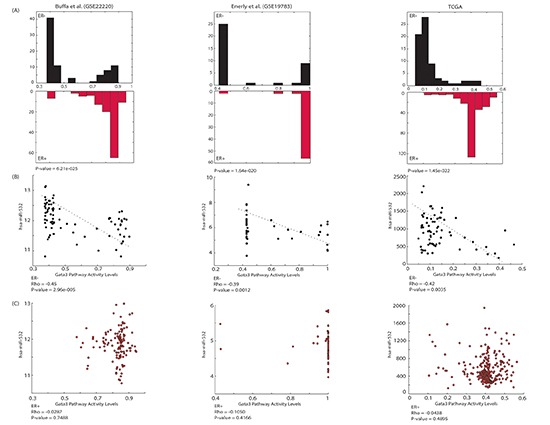
GATA3 pathway and hsa-miR-532 are associated selectively according to ER status **(A)** GATA3 pathway activity level distributions in the ER- and the ER+ groups of patients in three independent datasets. As the figure demonstrates, the pathway is more active in the ER+ group as opposed to the ER- patients. **(B)** A significant negative correlation between the pathway and the miRNA is observed in ER- patients and is **(C)** absent from the ER+ group. The correlation was calculated between the miR expression levels and the pathway activity levels. Correlation between the miR and the pathway may indicate on a possible control mechanism (ER- patients), while a lack of correlation (such in the ER+ group of patients) may implies on a broken control.

Using the same procedure we previously describe [23, 33], we demonstrate that the pathway *“GATA3 Participate in Activating the Th2 Cytokine Genes Expression”* demonstrated significantly higher activity levels in ER+ patients compared to ER- patients, across all three datasets, as can be seen from Figure 3(A).

Gata3 has previously shown to over-express in ER+ patients, and it has also suggested to regulate genes critical to the hormone-responsive breast cancer phenotype [34–36]. However, here we are referring to the pathway called Gata3 containing 13 different genes. Further, using the approach described above, we identified hsa-miR-532 as significantly targeting this GATA3 pathway. Specifically, hsa-miR-532 targets 6 out of the 13 genes in the pathway, leading to a *p*-value of 3×10^−5^. By separately obtaining the correlation of the miRNA and the pathway in the two clinical groups - ER+ and ER-, we found a consistent negative correlation between the miRNA and the pathway in the ER- group of patients, in all three datasets; and, we found (close to) zero correlation between the miRNA and the GATA3 pathway in the ER+ group of patients, as can be seen in Figure 3(B) and 3(C). These results are consistent with the previously known fact that the gene Gata3 is highly expressed in ER+ tumors. This observation may be explained by the finding we show here, a ‘broken’ control mechanism between Gata3 pathway and hsa-miR-532 in ER+ patients.

**Figure 4:**
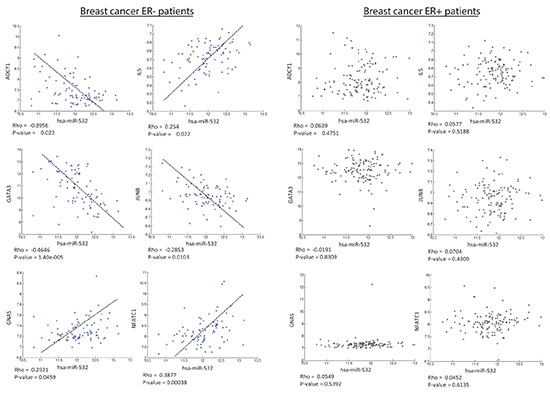
Predicted gene targets in the GATA3 pathway display correlation with hsa-miR-532 in the ER- group and not in the ER+ group Six genes in the Gata3 signaling pathway were found to have a possible binding site with hsa-miR-532. The graphs presented here show the correlation between miR-532 and the six genes in the two groups tested (ER- and ER+) in the TCGA dataset. As can be seen here, a significant correlation was found in the ER- patients while a no correlation was observed in the ER+ group.

When we observe the set of pathway genes, predicted to be targeted by hsa-miR-532, we see the behavior presented in Figure 4. In the figure, we see the six genes that were predicted to be targeted by hsa-miR-532 indeed correlate with miR-532 in the ER- group, while they are not correlated with the miRNA in the ER+ group. Three out of the six genes in the GATA3 pathway positively correlate with miR-532. Previous works have shown how different miRNAs induce (and not reduce) gene expression [37–40]. All genes in the pathway have the same expression distribution between the two groups (ER+ and ER-) except for the Gata3 gene, as can be seen in supplementary Figure 2. Nevertheless, the difference in the correlation status of the six genes that were identified as possible targets and hsa-miR-532 between the groups is still present.

In conclusion, these findings point to possible control mechanisms, involved with regulation in ER- breast cancer patients. As demonstrated, these results are consistent and robust in their presentation of the GATA3 pathway and its connection to hsa-miR-532. Further, the finding that this association is absent from ER+ tumors, suggests a possible intervention mechanism and calls for further study of a possibility of realigning regulation in breast cancer, from the harder-to-treat ER- phenotype, to the improved prognosis of ER+ phenotype.

### Validation in independent data sets

**Figure 5:**
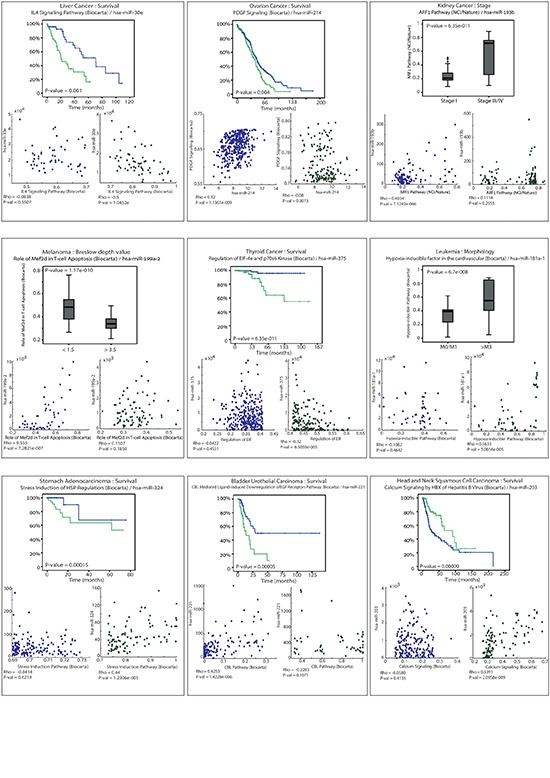
Results from nine different cancer datasets from the TCGA The described results were applied in nine different types of cancer in order to determine the strength of the presented results. For every type of cancer we identified a single pathway targeted by a single microRNA. In each cancer type the association between the miRNA and pathway is correlated with a specific phenotype. This phenomenon is consistent across a set of nine different cancer types.

The hypothesis of whole pathway regulation by miRNAs, through the multiple targeting of mRNAs of the same cellular pathway, mandates validation beyond the originating datasets. To validate whether the phenomenon may be associated with phenotype, we applied the same pipeline described above to the most comprehensive currently obtainable dataset. As the requirements for this dataset include miRNA and mRNA expression levels, as well as well documented phenotypic affiliation, we were able to combine information from patients in studies of unrelated nine different types of cancer. These data sets were downloaded from TCGA: ovarian, liver, melanoma, kidney, thyroid, leukemia, stomach adenocarcinoma, bladder urothelial carcinoma and head and neck squamous cell carcinoma. For each of these tumor types we downloaded, from TCGA, gene expression information to test different phenotypes. The following list of phenotypes is the ones tested and are available in TCGA database: survival, breslaw depth value, stage and morphology. Iterating over pathways as described above, we identified, for every one of the datasets, a single pathway that stratified the patients. Then, for each of these pathways, through the enrichment method described above, we identified a single microRNA that targets the identified pathway in a specific manner (results shown in Table 1 and Figure 5). These results support the hypothesis of a regulation mechanism directing pathway behavior through a miRNA association. The overarching results, over tumor types, phenotypes and studies, indicate that these phenomena may be at the core of cancer cellular mechanisms.

**Table 1:** The table presented here shows the results of the presented analyses in nine different datasets, every cancer show a specific pathway and a specific miRNA in a manner that correlates with different phenotype

Cancer Type	Phenotype	Pathway	microRNA
Ovarian serous cystadenocarcinoma	Survival	PDGF Signaling Pathway (Biocarta)	Hsa-miR-214
Liver hepatocellular carcinoma	Survival	IL4 Signaling Pathway (Biocarta)	hsa-miR-30e
Skin Cutaneous Melanoma	Breslow depth value	Role of Mef2d in T-cell Apoptosis (Biocarta)	hsa-miR-199a-2
Kidney renal clear cell carcinoma	Stage	ARF1 Pathway (NCI/Nature)	hsa-miR-193b
Thyroid carcinoma	Survival	Regulation of EIF-4e and p70s6 kinase (Biocarta)/	hsa-miR-375
Acute Myeloid Leukemia	Morphology	Hypoxia-inducible factor in the cardivascular system (Biocarta)	hsa-miR-181a-1
Stomach adenocarcinoma	Survival	Stress Induction of HSP Regulation (Biocarta)	hsa-miR-324
Bladder Urothelial Carcinoma	Survival	CBL Mediated ligand-induced downregulation of EGF receptors pathway (Biocarta)	hsa-miR-221
Head and Neck squamous cell carcinoma	Survival	Calcium Signaling by HBX of Hepatitis B Virus (Biocarta)	hsa-miR-203

## DISCUSSION

One possible major component involved with the reasoning of associating miRNAs and pathways is parallel gene expression. As every miRNA can potentially target hundreds of different transcripts simultaneously, by regulating the levels of a single miRNA, control over an entire pathway may be obtained. Previous studies have shown the vast target range of different miRNAs [41–46], but still, the specific processes and pathways regulated by individual miRNAs and their role in different diseases are mostly unknown. MiRNAs’ role in regulating cancer-related processes such as apoptosis, cell growth and tissue differentiation has been previously described and their key role in cancer is well established. In addition, cancer is a disease of multiple simultaneous modifications in genes. Altered global interactions evolve into the transformed and malignant states [47]. Oncogenesis gains an instructive perspective by being considered not only through a rearrangement of chromosomes but also as a rearrangement of regulatory networks.

Here, we suggest that a single pathway can be regulated by a single miRNA in a manner that ultimately directs phenotype. Results introduce a novel computational concept, the ability of a single miRNA to control cellular outcome via targeting of multiple genes in a specific pathway in a manner that ultimately directs phenotype. We demonstrate here, through the power of gene-expression networks, the criticality of miRNA-Pathway control mechanisms in driving disease course. By uncovering the specific interactions within the network, that are controlled by miRNAs and that drive the phenotype, we catalyze targeted treatment, facilitate prognosis through network biomarkers and offer a novel perspective into hidden disease heterogeneity.

## METHODS

### Hyper-geometric function analysis

Genes were matched to their corresponding pathways. The probability for the pathway being targeted by a specific microRNA was calculated using hyper-geometric function as follow:

$$\begin{array}{c} X_{i}-\;Number\;of\;targeted\;genes\;in\;pathway\;i\\ K_{j}-\;Number\;of\;targets\;found\;for\;miRNA\;j\\ N_{i}-\;Number\;of\;genes\;in\;pathway\;i\\ M-\;Number\;of\;total\;genes\;tested\\ \begin{array}{c} p_{j}=F(x|M,K,N)=1-\sum_{i=0}^{x}\frac{\left(\begin{array}{c} K\\ i \end{array}\right)\left(\begin{array}{c} M-K\\ N-i \end{array}\right)}{\left(\begin{array}{c} M\\ N \end{array}\right)} \end{array} \end{array}$$

The result is the probability of hitting up to x of possible K genes in N drawings.

For example: Gata3 signaling pathway comprises 13 genes (*N* = 13), six of them potentially targeted by hsa-miR-532 (*X* = 6). hsa-miR-532 was predicted to target 124 genes within the dataset (*K* = 124). Given that the total number of genes in the dataset is 1460 the resulted *p*-value for targeting Gata3 signaling pathway by hsa-miR-532 is 3 × 10^−5^.

### Pathway network interactions dataset

Network information was obtained from the National Cancer Institute's Pathway Interaction Database [19].

### Datasets

The Cancer Genome Atlas (TCGA): Breast cancer dataset and all datasets used for validation on different types of cancer were obtained from the TCGA database, available at http://cancergenome.nih.gov/ BRCA dataset holds molecular characterization of 322 BRCA patients. For each patient, the database provides gene expression microarray, microRNA values, and clinical information.

Gene Expression Omnibus (GEO): Two additional datasets were used in the breast cancer analysis GSE22220 [48] and GSE19783 [49].

### MicroRNA binding site prediction

miRWalk [20] is a comprehensive database that provides information on predicted and validated miRNA binding sites. It combines information produced by 8 established miRNA targets prediction programs: Diana-microT [50], miRanda [51], miRDB [52], PICTAR [53], PITA [54], RNA22, RNAhybrid [55], and Targetscan [56].

### Statistical and data analysis

Tow sample Student's T-Test was performed in order to estimate the pathways significance in stratifying phenotypes. Survival analysis was performed using Kaplan-Meier through clinical data to determine the power of a pathway in survival stratification.

### Random re-sampling

To determine the strength and accuracy of the overall performance of the miRNA-pathway association using bootstrapping, we scrambled the data and randomly build 357 new pathways with a random size between 4–50 genes (as in the original data). We then performed (as described) a prediction of microRNA binding sites in order to choose the most significant microRNA that associates with each random pathway and calculated *p*-value for all 357 microRNA-pathway pairs using hyper-geometric function. We iteratively repeated this analysis 1000 times. Figure 1(B) demonstrates the observed miRNA-pathway association distribution as opposed to the randomly build PPI-network.

### Networks construction

Networks generation performed using MATLAB R2010a and networks visualization created using Cytoscape [57, 58]. Every node represents either a pathway or a micro-RNA, edges represents the association between a micro-RNA and a pathway.

## SUPPLEMENTARY FIGURES AND TABLE





## References

[1] Calin GA, Croce CM (2006). MicroRNA signatures in human cancers. Nature reviews Cancerx.

[2] van Kouwenhove M, Kedde M, Agami R (2011). MicroRNA regulation by RNA-binding proteins and its implications for cancer. Nature reviews Cancer.

[3] Negrini M, Ferracin M, Sabbioni S, Croce CM (2007). MicroRNAs in human cancer: from research to therapy. J Cell Sci.

[4] Croce CM (2009). Causes and consequences of microRNA dysregulation in cancer. Nature reviews Genetics.

[5] Scott GK, Goga A, Bhaumik D, Berger CE, Sullivan CS, Benz CC (2007). Coordinate suppression of ERBB2 and ERBB3 by enforced expression of micro-RNA miR-125a or miR-125b. J Biol Chem.

[6] Cimmino A, Calin GA, Fabbri M, Iorio MV, Ferracin M, Shimizu M, Wojcik SE, Aqeilan RI, Zupo S, Dono M, Rassenti L, Alder H, Volinia S, Liu CG, Kipps TJ, Negrini M (2005). miR-15 and miR-16 induce apoptosis by targeting BCL2. Proc Natl Acad Sci U S A.

[7] Takamizawa J, Konishi H, Yanagisawa K, Tomida S, Osada H, Endoh H, Harano T, Yatabe Y, Nagino M, Nimura Y, Mitsudomi T, Takahashi T (2004). Reduced expression of the let-7 microRNAs in human lung cancers in association with shortened postoperative survival. Cancer Res.

[8] Kasinski AL, Slack FJ (2011). Epigenetics and genetics. MicroRNAs en route to the clinic: progress in validating and targeting microRNAs for cancer therapy. Nature reviews Cancer.

[9] Si ML, Zhu S, Wu H, Lu Z, Wu F, Mo YY (2007). miR-21-mediated tumor growth. Oncogene.

[10] Winter J, Jung S, Keller S, Gregory RI, Diederichs S (2009). Many roads to maturity: microRNA biogenesis pathways and their regulation. Nature cell biology.

[11] Lima RT, Busacca S, Almeida GM, Gaudino G, Fennell DA, Vasconcelos MH (2011). MicroRNA regulation of core apoptosis pathways in cancer. Eur J Cancer.

[12] Fu LL, Wen X, Bao JK, Liu B (2012). MicroRNA-modulated autophagic signaling networks in cancer. The international journal of biochemistry & cell biology.

[13] Lu TX, Hartner J, Lim EJ, Fabry V, Mingler MK, Cole ET, Orkin SH, Aronow BJ, Rothenberg ME (2011). MicroRNA-21 limits *in vivo* immune response-mediated activation of the IL-12/IFN-gamma pathway, Th1 polarization, and the severity of delayed-type hypersensitivity. J Immunol.

[14] Fang Y, Xue JL, Shen Q, Chen J, Tian L (2012). MicroRNA-7 inhibits tumor growth and metastasis by targeting the phosphoinositide 3-kinase/Akt pathway in hepatocellular carcinoma. Hepatology.

[15] Kefas B, Godlewski J, Comeau L, Li Y, Abounader R, Hawkinson M, Lee J, Fine H, Chiocca EA, Lawler S, Purow B (2008). microRNA-7 inhibits the epidermal growth factor receptor and the Akt pathway and is down-regulated in glioblastoma. Cancer research.

[16] Saydam O, Shen Y, Wurdinger T, Senol O, Boke E, James MF, Tannous BA, Stemmer-Rachamimov AO, Yi M, Stephens RM, Fraefel C, Gusella JF, Krichevsky AM, Breakefield XO (2009). Downregulated microRNA-200a in meningiomas promotes tumor growth by reducing E-cadherin and activating the Wnt/beta-catenin signaling pathway. Molecular and cellular biology.

[17] Glass C, Singla DK (2011). MicroRNA-1 transfected embryonic stem cells enhance cardiac myocyte differentiation and inhibit apoptosis by modulating the PTEN/Akt pathway in the infarcted heart. American journal of physiology Heart and circulatory physiology.

[18] Ponomarev ED, Veremeyko T, Barteneva N, Krichevsky AM, Weiner HL (2011). MicroRNA-124 promotes microglia quiescence and suppresses EAE by deactivating macrophages via the C/EBP-alpha-PU.1 pathway. Nature medicine.

[19] Schaefer CF, Anthony K, Krupa S, Buchoff J, Day M, Hannay T, Buetow KH (2009). PID: the Pathway Interaction Database. Nucleic acids research. (Database issue).

[20] Dweep H, Sticht C, Pandey P, Gretz N (2011). miRWalk—database: prediction of possible miRNA binding sites by “walking” the genes of three genomes. Journal of biomedical informatics.

[21] Storey JD (2002). A direct approach to false discovery rates. J Roy Stat Soc B.

[22] Efroni S, Schaefer CF, Buetow KH (2007). Identification of key processes underlying cancer phenotypes using biologic pathway analysis. PloS one.

[23] Ben-Hamo R, Efroni S (2012). Correction: Gene expression and network-based analysis reveals a novel role for hsa-miR-9 and drug control over the p38 network in glioblastoma multiforme progression. Genome medicine.

[24] Ben-Hamo Rotem ES (2013). Network as biomarker: Quantifying transcriptional co-expression to stratify cancer clinical phenotypes. Systems Biomedicine.

[25] Greenblum SI, Efroni S, Schaefer CF, Buetow KH (2011). The PathOlogist: an automated tool for pathway-centric analysis. BMC bioinformatics.

[26] Gruvberger S, Ringner M, Chen Y, Panavally S, Saal LH, Borg A, Ferno M, Peterson C, Meltzer PS (2001). Estrogen receptor status in breast cancer is associated with remarkably distinct gene expression patterns. Cancer research.

[27] Charpentier AH, Bednarek AK, Daniel RL, Hawkins KA, Laflin KJ, Gaddis S, MacLeod MC, Aldaz CM (2000). Effects of estrogen on global gene expression: Identification of novel targets of estrogen action. Cancer research.

[28] Bjornstrom L, Sjoberg M (2005). Mechanisms of estrogen receptor signaling: convergence of genomic and nongenomic actions on target genes. Mol Endocrinol.

[29] Leu YW, Yan PS, Fan M, Jin VX, Liu JC, Curran EM, Welshons WV, Wei SH, Davuluri RV, Plass C, Nephew KP, Huang TH (2004). Loss of estrogen receptor signaling triggers epigenetic silencing of downstream targets in breast cancer. Cancer research.

[30] Osborne CK (1998). Steroid hormone receptors in breast cancer management. Breast cancer research and treatment.

[31] Pietras RJ, Arboleda J, Reese DM, Wongvipat N, Pegram MD, Ramos L, Gorman CM, Parker MG, Sliwkowski MX, Slamon DJ (1995). HER-2 tyrosine kinase pathway targets estrogen receptor and promotes hormone-independent growth in human breast cancer cells. Oncogene.

[32] Warner M, Gustafsson JA (2010). The role of estrogen receptor beta (ERbeta) in malignant diseases—a new potential target for antiproliferative drugs in prevention and treatment of cancer. Biochemical and biophysical research communications.

[33] Ben-Hamo R, Efroni S (2012). Biomarker robustness reveals the PDGF network as driving disease outcome in ovarian cancer patients in multiple studies. BMC systems biology.

[34] Asselin-Labat ML, Sutherland KD, Barker H, Thomas R, Shackleton M, Forrest NC, Hartley L, Robb L, Grosveld FG, van der Wees J, Lindeman GJ, Visvader JE (2007). Gata-3 is an essential regulator of mammary-gland morphogenesis and luminal-cell differentiation. Nature cell biology.

[35] Tominaga N, Naoi Y, Shimazu K, Nakayama T, Maruyama N, Shimomura A, Kim SJ, Tamaki Y, Noguchi S (2012). Clinicopathological analysis of GATA3-positive breast cancers with special reference to response to neoadjuvant chemotherapy. Annals of oncology : official journal of the European Society for Medical Oncology / ESMO.

[36] Hoch RV, Thompson DA, Baker RJ, Weigel RJ (1999). GATA-3 is expressed in association with estrogen receptor in breast cancer. International Journal of Cancer.

[37] Place RF, Li LC, Pookot D, Noonan EJ, Dahiya R (2008). MicroRNA-373 induces expression of genes with complementary promoter sequences. Proceedings of the National Academy of Sciences of the United States of America.

[38] Ma F, Liu X, Li D, Wang P, Li N, Lu L, Cao X (2010). MicroRNA-466l upregulates IL-10 expression in TLR-triggered macrophages by antagonizing RNA-binding protein tristetraprolin-mediated IL-10 mRNA degradation. J Immunol.

[39] Tanaka H, Sasayama T, Tanaka K, Nakamizo S, Nishihara M, Mizukawa K, Kohta M, Koyama J, Miyake S, Taniguchi M, Hosoda K, Kohmura E (2013). MicroRNA-183 upregulates HIF-1alpha by targeting isocitrate dehydrogenase 2 (IDH2) in glioma cells. Journal of neuro-oncology.

[40] Ye W, Qin F, Zhang J, Luo R, Chen HF (2012). Atomistic mechanism of microRNA translation upregulation via molecular dynamics simulations. PloS one.

[41] Guo AY, Sun J, Jia P, Zhao Z (2010). A novel microRNA and transcription factor mediated regulatory network in schizophrenia. BMC systems biology.

[42] Yuan X, Liu C, Yang P, He S, Liao Q, Kang S, Zhao Y (2009). Clustered microRNAs’ coordination in regulating protein-protein interaction network. BMC systems biology.

[43] Xu J, Wong C (2008). A computational screen for mouse signaling pathways targeted by microRNA clusters. RNA.

[44] Ulitsky I, Laurent LC, Shamir R (2010). Towards computational prediction of microRNA function and activity. Nucleic acids research.

[45] Qiu C, Wang J, Cui Q (2011). miR2Gene: pattern discovery of single gene, multiple genes, and pathways by enrichment analysis of their microRNA regulators. BMC systems biology.

[46] Nam S, Kim B, Shin S, Lee S (2008). miRGator: an integrated system for functional annotation of microRNAs. Nucleic acids research.

[47] Green JE, Desai K, Ye Y, Kavanaugh C, Calvo A, Huh JI (2004). Genomic approaches to understanding mammary tumor progression in transgenic mice and responses to therapy. Clinical cancer research : an official journal of the American Association for Cancer Research.

[48] Buffa FM, Camps C, Winchester L, Snell CE, Gee HE, Sheldon H, Taylor M, Harris AL, Ragoussis J (2011). microRNA-associated progression pathways and potential therapeutic targets identified by integrated mRNA and microRNA expression profiling in breast cancer. Cancer research.

[49] Enerly E, Steinfeld I, Kleivi K, Leivonen SK, Aure MR, Russnes HG, Ronneberg JA, Johnsen H, Navon R, Rodland E, Makela R, Naume B, Perala M, Kallioniemi O, Kristensen VN, Yakhini Z (2011). miRNA-mRNA integrated analysis reveals roles for miRNAs in primary breast tumors. PloS one.

[50] Kiriakidou M, Nelson PT, Kouranov A, Fitziev P, Bouyioukos C, Mourelatos Z, Hatzigeorgiou A (2004). A combined computational-experimental approach predicts human microRNA targets. Genes & development.

[51] John B, Enright AJ, Aravin A, Tuschl T, Sander C, Marks DS (2004). Human MicroRNA targets. PLoS biology.

[52] Wang X (2008). miRDB: a microRNA target prediction and functional annotation database with a wiki interface. RNA.

[53] Krek A, Grun D, Poy MN, Wolf R, Rosenberg L, Epstein EJ, MacMenamin P, da Piedade I, Gunsalus KC, Stoffel M, Rajewsky N (2005). Combinatorial microRNA target predictions. Nature genetics.

[54] Kertesz M, Iovino N, Unnerstall U, Gaul U, Segal E (2007). The role of site accessibility in microRNA target recognition. Nature genetics.

[55] Rehmsmeier M, Steffen P, Hochsmann M, Giegerich R (2004). Fast and effective prediction of microRNA/target duplexes. RNA.

[56] Lewis BP, Shih IH, Jones-Rhoades MW, Bartel DP, Burge CB (2003). Prediction of mammalian microRNA targets. Cell.

[57] Saito R, Smoot ME, Ono K, Ruscheinski J, Wang PL, Lotia S, Pico AR, Bader GD, Ideker T (2012). A travel guide to Cytoscape plugins. Nature methods.

[58] Bastian M HS, Heymann S, Jacomy M (2009). Gephi: an open source software for exploring and manipulating networks. International AAAI Conference on Weblogs and Social Media.

