# Supplementary material for: MicroRNA regulation of molecular pathways as a generic mechanism and as a core disease phenotype
Source: Oncotarget. 2015 Jan 22;6(3):1594–604. doi: 10.18632/oncotarget.2734 (PMC4359317; doi:10.18632/oncotarget.2734)
Supplement: Supplementary file 1 [file oncotarget-06-1594-s001.pdf]

SUPPLEMENTARY FIGURES AND TABLE

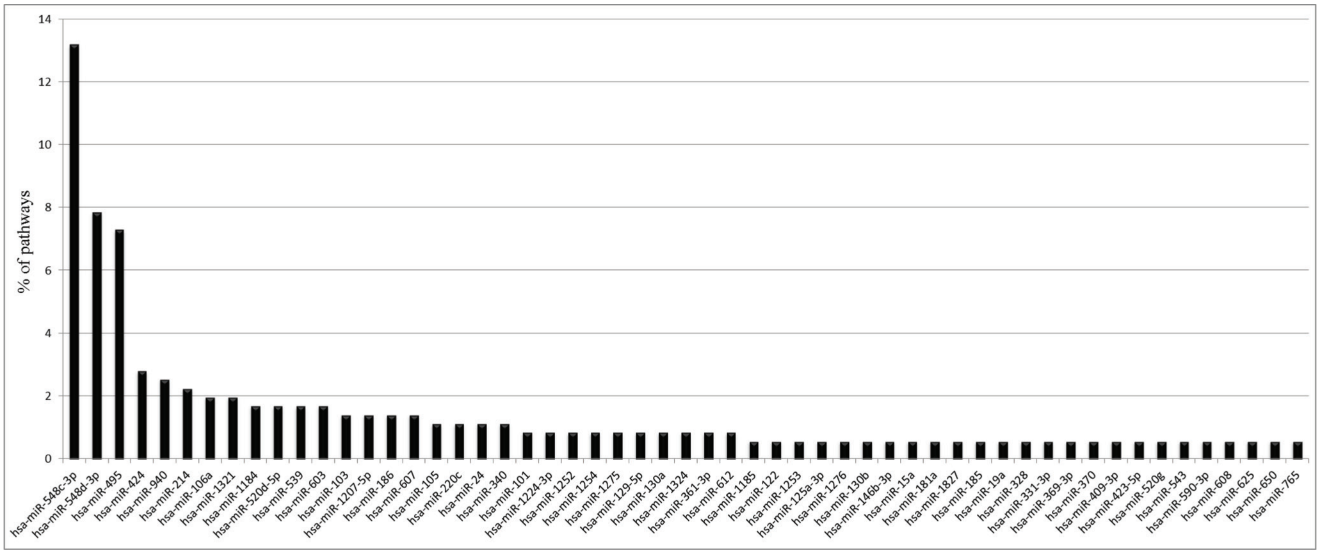

Supplementary Figure 1: microRNA distribution across all pathways. The presented graph highlight that specific microRNAs targets a wide spectrum of pathways.

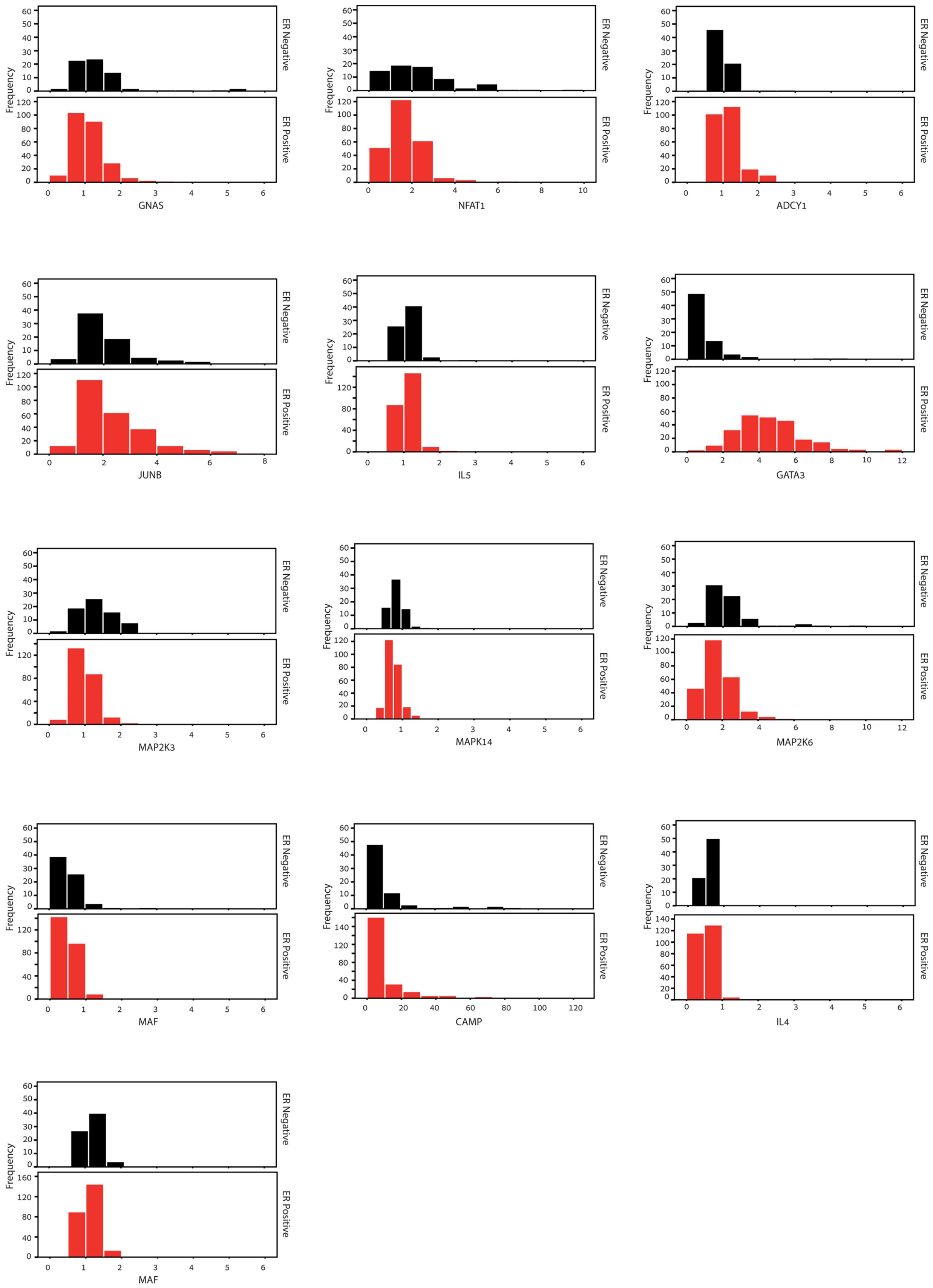

Supplementary Figure 2:

Supplementary Table 1: The table presented here shows the results of the microRNA-Pathways that were examined. In addition to the FDR adjusted *p*-value of each pair.
